# The impact of the COVID-19 pandemic on Canadian national team athletes’ mental performance and mental health: The perspectives of mental performance consultants and mental health practitioners

**DOI:** 10.3389/fpsyg.2022.937962

**Published:** 2022-08-18

**Authors:** Lori Dithurbide, Véronique Boudreault, Natalie Durand-Bush, Lucy MacLeod, Véronique Gauthier

**Affiliations:** ^1^School of Health and Human Performance, Dalhousie University, Halifax, NS, Canada; ^2^Faculté des Sciences de l’Activité Physique, University of Sherbrooke, Sherbrooke, QC, Canada; ^3^Department of Kinesiology, University of Ottawa, Ottawa, ON, Canada; ^4^Département de psychologie, Université du Québec à Trois-Rivières, Trois-Rivières, QC, Canada

**Keywords:** coronavirus, sport, high-performance, sport psychology, well-being

## Abstract

The COVID-19 global pandemic has led to significant disruptions in the lives of high-performance athletes, including the postponement of the Tokyo Olympic and Paralympic Games, the cancellation of many international and national competitions, and drastic changes in athletes’ daily training environment. The purpose of this research was to examine the interplay between the mental health and mental performance of Canadian national team athletes and the impact of the COVID-19 pandemic on these variables from the perspective of mental performance consultants and mental health practitioners. Twelve individuals working in these roles with national team athletes participated in focus groups and interviews during the second wave of the COVID-19 pandemic in Canada. Findings from the inductive reflexive thematic analysis revealed three main themes: (a) factors impacting athlete mental health (i.e., social and environmental, psychological, and public health restrictions), (b) consequences of COVID-19 for athletes (low mood symptoms, anxiety and stress symptoms, maladaptive behaviors, time for life outside of sport, rest, and recovery), and (c) impact of the pandemic on practitioners (roles, preparation and resources, gaps, and well-being). Interestingly, athletes with prior good mental performance skills were perceived to be more equipped to cope with challenges related to the pandemic, which concurrently seemed to facilitate good mental health throughout the pandemic. Furthermore, even though the pandemic had several debilitative consequences on athletes’ mental health, it imposed a break from training and competition that allowed them to rest and enjoy their life outside of sport. Finally, participants discussed the need for more mental health resources and better access to practitioners supporting mental performance and mental health in the Canadian sport system.

## Introduction

The COVID-19 global pandemic has led to significant disruptions in the lives of high-performance athletes, including the postponement of the Tokyo Olympic and Paralympic Games, the cancellation of many international and national competitions, and drastic changes in athletes’ daily training environment. In the initial months of the pandemic, many athletes and their National Sport Organizations (NSOs) shifted to different training strategies and programs, some of which have emphasized mental performance training. Further, increased attention was directed to the mental health of athletes as they navigated training and physical distancing constraints (e.g., no access to regular training facilities and resources, including teammates) and faced uncertainty about their sport career (NCAA Research, 2020; Reardon et al., 2021).

**Figure 1:**
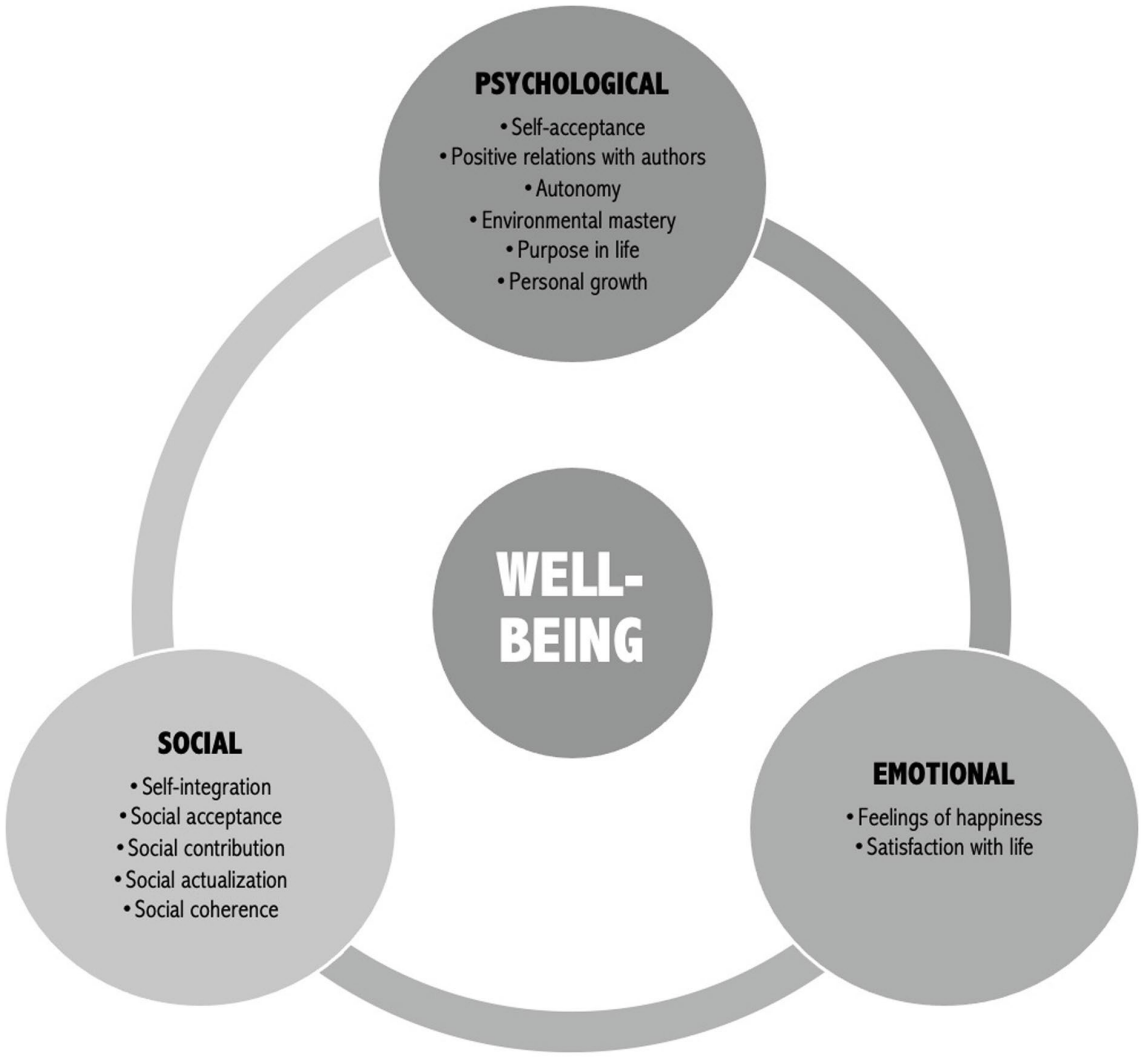
Dimension of psychological, emotional, and social well-being (Data extracted from Westerhof and Keyes, 2010).

Mental health has been an important focus in high-performance sport during the pandemic as it is recognized as a key component of elite athletes’ overall functioning and sport performance (Schinke et al., 2017; Durand-Bush and Van Slingerland, 2021). Mental health is characterized by a state of psychological, emotional, and social well-being in which individuals are capable to feel, think, and act in ways that allow them to enjoy life, realize their potential, cope with the normal stresses of life, work productively, and contribute to their community (Westerhof and Keyes, 2010; World Health Organization, 2014; see Figure 1). Mental health should not be confounded with mental illness, which is characterized by alterations in individuals’ feeling, thinking, and behaving, leading to significant distress, impaired functioning in their daily life, and diagnosable mental illnesses (e.g., anxiety and depression; World Health Organization, 2010).

Recent studies have shed light on the association between mental health and the stressors inherent in competitive sport participation (Reardon et al., 2019). Despite widely held assumptions that athletes are immune to mental health challenges because of their ability to pursue and excel at a high level, the reality is that high-performance athletes are as likely, and in some cases more likely, to experience compromised mental health (Moesch et al., 2018; Reardon et al., 2019; Åkesdotter et al., 2020). The public disclosure of mental health issues of high-profile athletes such as Michael Phelps, Simone Biles, and Carey Price, and emerging research on mental health in sport have shed light on the role of sport-specific risk factors in the onset and maintenance of mental health issues (Reardon et al., 2019; Henriksen et al., 2020). These factors are individual (e.g., sports injuries, performance perfectionism, and female athlete triad), social (e.g., dysfunctional relationship with teammates and coaches, violence, and abuse), and environmental (e.g., centralization, low income) in nature (Kuettel and Larsen, 2020). In addition, some dangerous or unhealthy practices (e.g., playing while injured and extreme weight control behaviors) can be encouraged or legitimized in some sport disciplines for the sake of performance, which can impair mental health (Reardon et al., 2019). For example, esthetic, endurance and weight class sports in which weight has a bearing on performance have been associated with disordered eating and eating disorders (Torstveit et al., 2008; Boudreault et al., 2021). Moreover, stigma related to mental illness is especially strong in elite sport cultures and can contribute to the maintenance and aggravation of mental health challenges and the reticence to seek help (Rice et al., 2016; Henriksen et al., 2020).

Studies conducted during the COVID-19 pandemic show that the mental health of elite athletes was impaired during this time (e.g., Kaya Ciddi and Yanzgan, 2020; NCAA Research, 2020; Ivarsson et al., 2021; Pensgaard et al., 2021). For example, in a study conducted with 379 Olympic and Paralympic Norwegian athletes, depression and insomnia were the most prevalent symptoms reported by athletes (Pensgaard et al., 2021). In this investigation as well as others, factors such as social isolation, being away from the team environment, reduced exercise, financial concerns, and uncertainty about the future were associated with decreased well-being (Kaya Ciddi and Yanzgan, 2020; Pensgaard et al., 2021). Olympic and Paralympic athletes perceiving positive consequences related to the COVID-19 lockdown showed a lower risk of mental health difficulties (Pensgaard et al., 2021). Some evidence also suggests that some athletes benefitted from the COVID-19 situation as it provided an opportunity to gain self-awareness (Tingaz, 2020) and recover from injuries (Jukic et al., 2020).

While it is fundamental to recognize that mental health challenges and mental illness symptoms are prevalent among elite athletes and that sport-specific factors can contribute to these manifestations, it is equally important to acknowledge the opportunities in elite sports to develop mental performance skills that can lead to positive outcomes (Durand-Bush and Van Slingerland, 2021). Mental performance reflects individuals’ proficiency with which they use mental skills to manage their thoughts, emotions, and behaviors to perform, reach their goals, and adapt to the demands of their changing environment (Durand-Bush and Van Slingerland, 2021). It involves cognitive processes such as attention, decision-making, perception, memory, reasoning, and coordination. It also encompasses key self-regulatory competencies such as goal-setting, planning, motivation, self-confidence, arousal/emotional/attentional control, imagery, self-talk, stress management, and evaluation (Durand-Bush et al., 2022). Just as high-performance athletes work to enhance their physical and technical skills, they should also spend a significant amount of their training strengthening the mental skills required to be successful in their sport (Calmels et al., 2003).

The Gold Medal Profile for Sport Psychology (GMP-SP) is a recent framework depicting 11 mental performance competencies deemed essential to succeed in high-performance sport (Durand-Bush et al., 2022). The model integrates fundamental competencies (i.e., motivation, confidence, and resilience), self-regulation competencies (i.e., self-awareness, stress management, emotion and arousal regulation, and attentional control), and interpersonal competencies (i.e., athlete-coach relationship, leadership, teamwork, and communication). In this context, competencies refer to measurable patterns of knowledge, skill, abilities, behaviors, and other characteristics that individuals require to effectively perform their roles, functions, or tasks (Rodriguez et al., 2002). Of importance, the GMP-SP highlights the interplay between mental performance and mental health. Mental health is featured as an overarching construct that influences the achievement of mental and athletic performance in high-performance sports (Durand-Bush et al., 2022).

The level of mental performance of athletes can vary based on different factors such as access to a Mental Performance Consultant (MPC), the value placed on mental performance by coaches and teammates, and early career exposure to mental performance work (Weinberg and Williams, 2015). In Canada, MPCs work closely with national teams as a part of an integrated support team (IST) to help athletes reach their performance potential. They assist them in developing a variety of mental performance competencies based on personal and sport-specific needs (Durand-Bush et al., 2022). MPCs also play a role in promoting and fostering mental health, as highlighted by Durand-Bush and Van Slingerland (2021).

MPCs are in an ideal position to recognize and respond to athletes who are struggling with their mental health. MPCs may be the trusted individuals to whom athletes disclose their challenges. In other cases, they may be the ones within athletes’ integrated support teams noting significant shifts in their behaviors, appearance, and overall functioning, suggesting that they may be in need of additional support (p. 84).

Athletes with compromised mental health may not be able to optimize their mental performance, including their ability to pay attention, plan, make sound decisions, solve problems, and store and retrieve information from memory (Trivedi, 2006; Keshavan et al., 2014). This highlights the importance of considering both mental performance and mental health when attempting to understand the functioning of athletes. Unfortunately, limited research has integrated both constructs, which limits our grasp of how MPCs and mental health practitioners can be best leveraged to support athletes during atypical and stressful times like the COVID-19 pandemic.

Considering the stressors and disruptions to which elite athletes were subjected during the pandemic and evidence showing that mental performance and mental health are intertwined (e.g., Schinke et al., 2017; Durand-Bush and Van Slingerland, 2021), it is important to examine if athletes’ mental performance skills impacted their perceptions of and responses to the pandemic. It would also be valuable to know to what extent athletes sought psychological support from MPCs and mental health practitioners during the pandemic. While a few studies have been conducted with athletes to examine how they have navigated COVID-19 (e.g., Kaya Ciddi and Yanzgan, 2020; Ivarsson et al., 2021; Pensgaard et al., 2021), more research is warranted and the perspectives of practitioners supporting athletes should be addressed as they have not been the focus of studies thus far and they may shed additional light on the interplay between mental performance and mental health.

The purpose of this research was to fill the aforementioned gap and examine the interplay between the mental health and mental performance of Canadian national team athletes and the impact of the COVID-19 pandemic on these variables. Given the role that MPCs and mental health practitioners played in providing support to Canadian national team athletes during the pandemic, and their interactions and work with multiple and diverse athlete populations, they were the focus of this investigation.

The specific objectives and research questions were:

Objective 1: Examine the interplay between the COVID-19 pandemic and mental health.

1. Did athletes maintain their mental health during the pandemic and did their pre-pandemic mental health influence their responses to the pandemic and their ability to manage their mental health throughout?
2. What positively and negatively impacted athletes’ mental health during the pandemic?
3. What role did MPCs and mental health practitioners play in supporting athletes’ mental health during the pandemic?

Objective 2: Examine the interplay between the COVID-19 pandemic and mental performance.

1. Did athletes’ pre-pandemic mental performance skills influence their responses to the pandemic and their ability to self-regulate throughout?
2. Did athletes use the pandemic as an opportunity to work on their mental performance skills?
3. What role did MPCs and mental health practitioners play in supporting athletes’ mental performance during the pandemic?

The study was carried out during Canada’s second wave of the COVID-19 pandemic when several restrictions were in place to prevent further spreading of the virus. Regulations were dictated by each province and territory, and consequently varied significantly depending on where participants lived. For instance, while some participants were supporting athletes who were isolated at home and trained on their own, others worked with athletes who were allowed to train with others within a “bubble” environment. Furthermore, regulations evolved during the course of this study, which impacted both the participants and the sport community they served. It is important to consider this contextual information when interpreting the results of this research.

## Materials and methods

### Research team

The research team involved three MPCs who work in high-performance sport and have an academic position at three different Canadian universities. One of the MPCs is also a clinical psychologist. All three individuals have experience addressing both mental performance and mental health in their consulting practice and research. The team also involved two graduate students—one who is completing a psychiatry program and was a former Canadian national team athlete (i.e., spring kayak) and the other who is completing a clinical psychology program and is a varsity student-athlete.

### Participants

The sample included a total of 12 practitioners (9 female; 3 male), of which seven were MPCs, one was a mental health practitioner, and four had dual credentials (MPC and mental health practitioner). MPCs were professional members of the Canadian Sport Psychology Association and mental health practitioners were either a registered psychologist or a certified clinical counselor in Canada (see Table 1). Participants worked with at least one Canadian national/Olympic/Paralympic team, and in some cases, with multiple teams and athletes. Overall, over 30 Canadian sports/teams were represented, including summer and winter sport teams and both men’s and women’s teams.

**Table 1:** Participant descriptives.

Participant #	Credentials	Region	Gender
1	MHP, MPC	Central	Female
2	MPC	Central	Female
3	MPC	Western	Female
4	MHP, MPC	Western	Female
5	MPC	Central	Male
6	MPC	Western	Male
7	MHP, MPC	Western	Female
8	MPC	Western	Female
9	MHP, MPC	Western	Female
10	MPC	Atlantic	Male
11	MHP	Central	Female
12	MPC	Atlantic	Female

MPC, Mental performance consultant; MHP, Mental health practitioner; Region: Western = British Columbia & Alberta, Central = Saskatchewan, Manitoba, Ontario, Quebec, Atlantic = Nova Scotia, New Brunswick, Prince Edward Island, Newfoundland and Labrador.

Participants were recruited through a network of approximately 40 MPCs and mental health practitioners who work with and support Canadian national team athletes. Invitations to participate in the study were forwarded to the network *via* email and follow-up announcements were made at monthly meetings. Participants were directed to email one of the researchers to schedule a time to participate in a focus group interview. Further, once focus group interviews were scheduled with at least two participants, follow-up emails to the network were sent to invite other practitioners to join. The recruitment strategy generated three focus groups and two participants completed separate individual interviews, as they could not accommodate the focus group times into their schedules.

### Data collection

The data were collected through focus group and individual interviews and were conducted in English by the same researcher (i.e., graduate student in psychiatry research program and former national team athlete) with advanced interviewing and counseling skills, and high-performance sport knowledge. The focus groups permitted dialog among participants and allowed the researcher to gain a broad understanding of experiences while drawing attention to specific examples and nuances within the participants’ shared world. While focus groups are often used as a preliminary step in exploratory research, they can also stand alone as a research method to explore a new area of study or an unfamiliar topic (Wilkinson, 1998). Given the novelty of this investigation on the impact of COVID-19, focus groups were deemed suitable to allow for the identification of both individual and collective perspectives and experiences in an interactive and efficient manner. The focus group and individual interviews lasted an average of 90 and 120 min, respectively. The same semi-structured interview guide was used for all interviews to ensure that the research questions were adequately addressed. However, the guide was flexible and promoted responsive interviewing (i.e., continuous give-and-take between the researcher and respondents to maintain direction without overbearing structure; Rubin and Rubin, 2012). All interviews were recorded, transcribed verbatim, and occurred between November 2020 and February 2021, which coincided with the second wave of the COVID-19 pandemic in Canada. Given that public health is governed at the provincial/territorial level, policies and restrictions varied across the country, which may have impacted participants’ views and experiences.

### Data analysis

An inductive reflexive thematic analysis was performed by two of the researchers to thoroughly describe and understand the data while also considering the context of the phenomenon under study (i.e., COVID-19). The analysis was guided by Braun and Clarke’s (2006, 2019) recommendations: (a) immerse oneself in the data for deep familiarization; (b) record initial ideas, comparisons, and reactions; (c) import transcripts and researcher notes into NVivo software; (d) code the data and group codes into high-order themes; and (e) work reflexively throughout the development and organization of themes to question and re-negotiate definitions and interpretations of the dataset.

In addition, the two researchers completed training together to ensure that they used a congruent approach and followed the same steps. For instance, they coded the data using similar terminology based on the research questions while remaining flexible to use terms most representative of the data. They communicated regularly, compared their interpretations, and collaborated to code the more ambiguous data. To enforce qualitative rigor, the thematic structure (i.e., name and hierarchy of codes and content categorized under these codes) was checked by “critical friends” (Smith and McGannon, 2018), through a series of formal meetings with the three other members of the research team, who are experienced in the field. This process facilitated the interpretation of the data in relation to existing empirical evidence and practices and consideration of alternative perspectives and feedback. The thematic structure went through several iterations before the team approved the final version.

## Results

Overall, the findings of the study produced three main themes: (a) factors impacting athlete mental health, (b) consequences of COVID-19 for athletes, and (c) impact of the pandemic on practitioners (see Tables 2–4). Each theme and sub-themes are addressed below, along with one or two citations to support findings.

**Table 2:** Factors impacting athlete mental health.

Sub-themes	Categories	Sub-categories	*N*
Factors impeding athlete mental health	Social and environmental	Isolation	9
		Stressors outside of sport	5
		Online communication fatigue	6
	Psychological	Fear of virus	8
		Loss of control	2
		Exacerbation of prior mental illness	4
		Identity crisis	5
		Uncertainty of future	6
		Inability to Transfer Mental Performance Skills	2
	Public health restrictions	Creation of disadvantages	5
		Disruption of daily routines	6
Factors facilitating athlete mental health	Social and environmental	Social support	8
		Communication	8
		Online resources	9
	Psychological	Mental skills taught by MPCs	12
		Good prior mental performance	11
		Good prior mental health	2
		Autonomy	3

**Table 3:** The consequences of COVID-19 for athletes.

Sub-themes	Categories	*N*
Debilitative consequences of COVID-19	Low mood symptoms	12
	Anxiety and stress symptoms	11
	Maladaptive behaviors	2
Facilitative consequences of COVID-19	Time for life outside of sport	4
	Rest and recovery	9

**Table 4:** The Impact of the pandemic on practitioners.

Sub-themes	Categories	*N*
Roles		12
Preparation and resources		10
Gaps	Mental health resources and procedures	7
	Greater awareness for mental health	3
	Coach/IST support	6
Well-being		10

### Context

It is important to first note the range of positive and negative impacts of the COVID-19 pandemic on athletes as perceived by our participants given the broad Canadian context and the different provincial/territorial regulations and restrictions imposed throughout the pandemic. One participant stated, “Obviously it varies from athlete to athlete. I can see some have been extremely negatively impacted” (p. 12). Other participants highlighted how the Paralympic athletes with whom they worked were able to cope with the pandemic: “In their recovery, [they] are able to be resilient, so they are […] a prepared, open, sound mind, helpful group who did not experience much trouble at all” (p. 2). On the other hand, one participant described greater challenges for Paralympic athletes, “You saw that people were getting in a more isolated situation, depending on the circumstances. Visually impaired athletes, for example, were not allowed to travel in any way. Not even allowed to use public transit” (p. 6).

Participants also reported differences between summer and winter athletes. Specifically, Participant 5 stated, “For winter sports, it’s been a little bit easier. The consensus we found with the coaches was to give them a long recovery time, which was beneficial to them. For summer sports, I had to face different reactions.” Whether or not athletes had qualified for the summer Olympic or Paralympic Games also seemed to influence athletes’ responses to the pandemic. Participant 5 stated, “Most of them were already qualified for the games, which was a safer situation, but it was a bit different for others.” Lastly, some athletes also played in professional leagues. Relying on income from professional sports may have created greater stress for some athletes. Participant 10 explained, “Most of our players live and support themselves and in some cases their families on the income they make playing professional… so that created a big stressor.”

### Theme 1: Factors impacting athlete mental health

The first main theme was divided into two sub-themes - “Factors impeding athlete mental health” and “Factors facilitating athlete mental health” - which were, in turn, divided into categories and sub-categories (see Table 2). The numbers in the right column indicate the number of participants who mentioned the topic, which give insight into the commonality and uniqueness of experiences.

#### Factors impeding athlete mental health

According to the participants interviewed in this study, Canadian national team athletes experienced some challenges with their mental health throughout the course of the pandemic. Several factors (i.e., social and environmental, psychological, and public health restrictions) contributed to these challenges.

##### Social and environmental

The social and environmental factors that hindered athletes’ mental health included isolation, stressors outside of sport, and online communication fatigue.

###### Isolation

Participants indicated that the isolation that athletes experienced, either in the initial lockdown or during the self-isolation required when traveling or when exposed to the virus, was a contributing factor to mental health challenges, particularly athletes’ social well-being. For example, Participant 4 said, “There’s a reason isolation is a form of torture. Being in a room for 14 days on your own, not in your home country is difficult for many individuals.”

###### Stressors outside of sport

For many athletes, the pandemic brought about additional non-sport-related stressors that impacted all dimensions of well-being. Not being able to visit family members, social justice causes (e.g., Black Lives Matter), and financial responsibilities unrelated to sport seemed to negatively affect some athletes. As Participant 7 stated, “One of our athletes has her own business and just that has caused a lot of stress.”

###### Online communication fatigue

Fatigue stemming from online communication, particularly when athletes returned to in-person training, was identified by participants as a debilitative factor. As Participant 6 described, “[…] then the zoom fatigue set in with the return to training, which then made it more difficult to have contact.” This state of depletion impacted athletes’ interactions (i.e., social well-being) and mental capacity (i.e., psychological well-being).

##### Psychological

Psychological factors that impeded athletes’ mental health included fear of the virus, loss of control, exacerbation of prior mental illness, identity crisis, uncertainty of the future, and inability to transfer mental performance skills outside of sport performance.

###### Fear of the virus

Participants indicated that many athletes feared the COVID-19 virus, which challenged emotions (i.e., emotional well-being) and relationships (i.e., social well-being). They were afraid of catching the virus and/or of being responsible for infecting others such as teammates or family members, as stated by Participant 2: “If, for example, you had limited lung function, certain athletes were more fearful of that [the virus] and return to play.”

###### Loss of control

Many athletes who were accustomed to highly structured and planned training programs and schedules also experienced a sense of loss of control (i.e., autonomy), which impacted their psychological well-being. Participant 12 noted, “If I were to take COVID as a whole and the team as a whole, there definitely has been a negative impact; they have felt a loss of control.”

###### Exacerbation of prior mental illness

Some athletes were living with a mental illness prior to the onset of the pandemic. According to the practitioners, these athletes were more negatively impacted than others as their symptoms were exacerbated during the pandemic. For example, Participant 1 stated, “Other athletes were more impacted right from the beginning, especially those who already suffered from anxiety or were dealing with depression right from the start.”

###### Identity crisis

Practitioners noted that some athletes experienced an identity crisis where they pondered their role in society, how essential they were perceived, and their life purpose, which has inevitable psychological and social well-being implications. For example, Participant 11 stated, “They were like ‘Ok, I’m an athlete, how do I contribute to society? Is it really useful what I’m doing? I’m not an essential service’ […] Those anxiety and existential questions kind of came through.”

###### Uncertainty of future

As a result of the postponement of the 2020 Games and the cancellation of other major international events, many athletes had to rethink their life plans both within and outside of sport. Participants noted that this created some challenges to their overall mental health. For example, Participant 7 stated, “The anxiousness was definitely around uncertainty and trying to make certain life decision […] like do I postpone my wedding? Do I say no to that grad school?”

###### Inability to transfer mental performance skills

While athletes had been working with MPCs before and during the pandemic to develop or strengthen their mental skills, not all athletes were able to transfer these skills from competitive to non-sport environments, which limited the optimization of their mental health. Participant 12 stated, “It seemed as though they did not adapt the skills to the situation very well. They have kept them for high performance competition, which is what they learned them for.”

##### Public health restrictions

Public health restrictions have been integral to the containment and management of the COVID-19 pandemic. However, these restrictions have been a notable factor causing challenges to athletes. The main sub-factors have been the creation of disadvantages and the disruption of daily routines.

###### Creation of disadvantages

Due to the differences in public health restrictions not only across different countries but also within Canada across each province/territory, athletes were required to follow different rules. For example, in some locations, training indoors was permitted, while in others, it was completely restricted. This created additional stress for some athletes, particularly leading up to the Tokyo Olympic and Paralympic Games, as Participant 4 stated, “These guys are allowed to do this and Canada is saying these are rules. That’s an unfair disadvantage that produces stress.” Such stressors particularly impacted athletes’ emotional (e.g., anxiety, frustration) and psychological well-being (e.g., low autonomy and environmental mastery).

###### Disruption of daily routines

According to practitioners, athletes had highly structured and busy schedules filled with training, competitions, traveling, and recovery prior to the pandemic. Many also pursued additional endeavors such as school, work, or sponsorships. With the implementation of public health restrictions, many of these activities came to a complete halt. Changes in regular routines created additional mental health challenges (e.g., lack of environmental mastery), as illustrated by Participant 6, “These are athletes who are used to a lot of activity, training, and competition. Some of them were more or less bouncing off the walls in their basement suites and in their apartments. They are not used to not doing a lot on a daily basis.”

#### Factors facilitating athlete mental health

While the previous factors were perceived to hinder athlete mental health, a series of factors were described as facilitative. Practitioners noted several social/environmental and psychological factors that provided opportunities to enhance rather than threaten athletes’ mental health.

##### Social and environmental

The social and environmental factors deemed facilitative to athlete mental health included social support, communication, and online resources.

###### Social support

Participants noted that a good support network both within and outside of sport facilitated athletes’ ability to cope with mental health challenges, particularly during the initial months of the pandemic. Participant 3 stated, “Having a really dialed in support network, as well as a peer network […] seemed to really assist them as they were going through these periods of self-isolation.” This fostered self-integration and social acceptance, which are fundamental to social well-being.

###### Communication

Athletes’ mental health was strengthened when there was open and clear communication. This could include communication regarding changes in schedules, plans, or restrictions, or discussions that simply brought people together virtually. For example, Participant 8 noted, “If there was communication, it was a relief, even if it was limited. I think [it is] better to have communication and sharing what we know and why instead of a lack thereof.” Further, Participant 2 stated, “Many of the early workshops I did were small group discussions on what they did to cope, so they were able to help each other a lot with sharing what they did,” which demonstrates the facilitative impact of communication for teamwork and all aspects of well-being.

###### Online resources

The provision of online resources helped to support athletes’ mental health. For example, Participant 12 stated, “I’ll send them a resource, like a podcast or a YouTube video for them to watch or listen.” Participant 10 noted some valuable online resources used by athletes to maintain their emotional and psychological well-being, “The Calm app and HeadSpace were used quite a bit by our athletes.” Having said this, it was important to temper this with online communication fatigue, as illustrated in a previous section.

##### Psychological

Several psychological factors were described as positive for athlete mental health and they encompassed mental skills taught by MPCs and mental health practitioners, good prior mental performance, good prior mental health, and autonomy.

###### Mental skills taught by MPCs and mental health practitioners

Participants noted that their services were requested at a much greater rate during the pandemic than before its onset. Mental performance training focused on skills required for competitive performance and coping with the pandemic such as stress management and emotional regulation, which facilitated athletes’ psychological and emotional well-being: “Stress and emotional regulation, and mindfulness practice. The self-awareness piece was huge in implementing goal setting and regulation practice” (p. 8).

###### Good prior mental performance

The work that athletes had completed years prior to the pandemic had a positive impact on their overall mental health during the pandemic. As Participant 9 noted, “We had done work already in year prior, not expecting a pandemic. Mindfulness work and gratitude work, and being in the moment.” Further, Participant 4 reported, “[The athletes] had some of the tools to regulate, they had the ability to navigate and tolerate uncertainty, they had the mindfulness skills to be able to regulate daily.” Participant 4 added, “The pre-existing foundation, their experience and tolerance in uncertainty, this is something that they had learned to do because they were in sport their whole life… in addition to the access to professional resources [they had] to help them manage that.”

###### Good prior mental health

Much like poor prior mental health was a debilitative factor, good prior mental health was a facilitative factor for overall athlete mental health during the pandemic. Participants noted that athletes who had a high level of emotional and psychological well-being prior to the pandemic seemed to cope better. For example, Participant 12 stated, “I would say athletes who did not show any signs of depressive mood or anxiety to begin with, did not seem to waver much with this.”

###### Autonomy

Participants also noted that autonomy positively contributed to athlete mental health. Allowing athletes to reach out on their own when necessary, as opposed to forcing this or imposing resources on them was deemed beneficial, as shown by Participant 8, “Letting [athletes] have the autonomy to reach out if that relationship is not there. But then just offering it too and creating that safe space if there is engagement.” Both autonomy and environmental mastery (e.g., ability to control the use of resources and opportunities available in the environment) are key dimensions of psychological well-being.

### Theme 2: Consequences of COVID-19 for athletes

The second main theme was divided into two sub-themes - “Debilitative consequences of COVID-19” and “Facilitative consequences of COVID-19” - which were, in turn, divided into categories (see Table 3). Although some of the consequences of the pandemic overlapped with certain aforementioned factors, it was important to discern what *contributed to* athlete mental health during the pandemic and what *consequences resulted from* the pandemic for both athletes and practitioners.

#### Debilitative COVID-19 consequences

Practitioners discussed several debilitative consequences of the COVID-19 pandemic related to athlete mental health, including low mood symptoms, anxiety and stress symptoms, and maladaptive behaviors. While mental performance and mental health practitioners were able to recognize mental health challenges and mental illness symptoms, only those with clinical training diagnosed and treated mental illness.

##### Low mood symptoms

Participants noted several symptoms in athletes that aligned with low mood, albeit this did not signify that they had a mental illness. In many cases, symptoms such as lack of motivation, loneliness, sadness, and fatigue, which are characteristics of low psychological, emotional, and social well-being, were expected given the number of debilitative factors with which athletes had to contend throughout the pandemic. For example, Participant 6 noted, “I’m seeing more of the COVID fatigue setting in, and difficulties with motivation,” while Participant 4 mentioned that “the fatigue and the feeling of being absolutely done” were experienced by athletes. Loneliness was another symptom, as discussed by Participant 4, “They’re certainly missing contact. I think that goes for all of them […] the inability to be with other people has been a significant problem.” Finally, some athletes experienced sadness, particularly when the postponement of the Tokyo Olympic and Paralympic Games was announced. Participant 7 explained, “When the Olympics were cancelled […] that’s when it hit another low.”

##### Anxiety and stress symptoms

Many athletes experienced increased levels of anxiety and stress with impacts on their emotional and psychological well-being, as described by Participant 7: “[There was] anxiety over uncertainty and anxiety over lack of routines and lack of familiarity. It gets their head spinning.” Further, Participant 3 stated, “Their stress increased, their mood fluctuations increased, uncertainty increased.” And Participant 6 added in reference to the initial Games postponement, “There was a lot of panic, stress and uncertainty when team Canada pulled out of the Tokyo Olympics, and with the postponement of the games and what that meant for them.”

##### Maladaptive behaviors

The pandemic also led to maladaptive behaviors in some athletes, compromising their well-being and triggering symptoms of mental illness. This included overtraining and indications of potential disordered eating. Participant 11 noted, “The athletes with eating disorders, the symptoms got exacerbated during COVID because of the lack of control about everything, which could have triggered their control of food.” Participant 7 added “We saw a lot of athletes hyper focus on, and perhaps over train during COVID.”

#### Facilitative COVID-19 consequences

Some athletes experienced facilitative consequences of the COVID-19 pandemic, favorably impacting their mental health. Practitioners observed that some athletes benefited from the pandemic by having more time for life outside of sport as well as for rest and recovery. Other athletes experienced relief and hope during the pandemic.

##### Time for life outside of sport

Lockdowns allowed athletes to spend more time with family and friends and participate in activities for which they typically did not have time prior to the pandemic, which facilitated their social well-being. Other athletes discovered new hobbies and were able to spend time at home during a period when they would typically be traveling. Participant 7 stated, “They were loving going biking for half the day and hiking. There was a lot of peace in that.” This had a positive effect on their emotional well-being.

##### Rest and recovery

The extra time off, particularly for summer athletes for whom the Olympic and Paralympic Games were postponed, was of great benefit to many athletes who took advantage to rest and recover. Particularly for those athletes who were dealing with injuries, the added time came as a break and opportunity to take things slower than initially anticipated. Participant 1 explained, “Some people felt [that quarantine] was coming on like a break that they needed for recovery and needed mentally. They were quite happy to have that break from training during the first wave.” Participant 3 highlighted the relief that some athletes felt as a result of this additional time for recovery: “[There was a] feeling of relief, they were tired. Then there was a big push and they kind of welcomed a bit of a break.” These examples demonstrate how their psychological and emotional well-being was bolstered.

### Theme 3: Impact of the pandemic on practitioners

The third main theme pertained to the impact of the pandemic on the practitioners themselves, which was divided into the four sub-themes of “roles,” “preparation and resources,” “gaps,” and “well-being” (see Table 4).

#### Roles

Participants noted that their roles shifted during the pandemic. Many became leaders within their integrated support teams. With athlete training and competition severely limited, there was also a shift in focus to overall health and well-being. For example, Participant 4 noted, “There is also a systemic role […] The psychosocial has not been addressed in all of these procedural things and so [there was a need for] efficacy, leadership, and alignment.” Moreover, Participant 3 explained, “So the nature of our work, the content of our work, had to shift and change. It had to go to emotional regulation. It had to go to coping strategies.”

#### Preparation and resources

Practitioners’ level of preparation to support athletes and others throughout the pandemic varied based on experience, training, and available resources. For instance, while some felt prepared as illustrated by Participant 7, “What I defaulted to was my grief work. Any grief work that I had done in studies and just that I’d had, I called on those,” others perceived to be less ready, “It’s an unprecedented event without a playbook” (Participant 4).

A number of resources from other fields helped practitioners to fulfill their roles: “A couple of resources came from the corporate world, the organizational psychology field. For example, articles, webinars on leading in times of crisis, taking care of employees […] all those things were helpful” (Participant 1). Participants also discussed being part of a valuable network of mental performance and mental health practitioners: “I was so thankful to have attended every one of the COVID-19 alignment sessions. We started meeting very frequently at the beginning […] and then as we returned to play, [it was] spaced out. It’s really good professional development” (Participant 3).

#### Gaps

Participants identified gaps within the sport system because of the pandemic such as the need for mental health resources and procedures, greater awareness for mental health by national sport organizations, and the need for coach and IST mental performance and mental health support.

##### Mental health resources and procedures

Most participants indicated a need for more mental health resources within the Canadian high-performance sport system. For instance, Participant 12 stated, “I think that sport in general needs to invest more in mental health and they will see a great return on investment.”

##### Greater awareness for mental health

Some national sport organizations (NSO) had a disregard or lack of awareness of the impact of mental health and its relevance. For example, Participant 7 noted, “The [NSO] culture is very much a dictatorship […] There was an email where the [staff] stated, ‘Suck it up […] you are lucky to be training right now given the situation’. Minimizing those feelings definitely made it worse.” Participant 8 added “Some programs may even not have an MPC available all the time, so when you are at trainings or when you are at camps […] there may be a limited accessibility factor in certain situations.”

##### Coach/IST support

Coaches and support staff also experienced both debilitative and facilitative consequences of the pandemic and participants noted the gap in mental performance and mental health support for these individuals. For example, Participant 2 noted the “stress that the medical staff is under because of the return to play. They’ve described it specifically as ‘I am all of a sudden an expert in something that nobody is an expert in.”

#### Well-being

Considering the increase in demand and recognition for their support and services, practitioners experienced challenges with their own well-being. They reported using the same coping skills they were teaching their athletes. Participant 7 highlighted, “Well I’m trying to practice what I preach. I would say I’m not a consistent meditator, but I am finding other ways to get to that space mentally.” Most participants reported experiencing some level of compassion fatigue, as summarized by Participant 1, “So the question is, who takes care of the caregiver? It’s been a long year.”

## Discussion

The purpose of this research was to examine the interplay between the mental health and mental performance of Canadian national team athletes and the impact of the COVID-19 pandemic on these variables. Specifically, we wanted to know if athletes’ mental health was impacted during the pandemic, if mental performance skills made a difference, if MPCs and mental health practitioners played a role in supporting mental health and mental performance, and if there were gaps in resources.

### Factors impacting athlete mental health

Our results suggest that athletes’ mental health (i.e., psychological, emotional, social well-being) was affected during the pandemic and both facilitative and debilitative factors were identified. In terms of positive factors, athletes’ mental health prior to the pandemic was influential. This finding makes sense given the nature of the COVID-19 crisis and the psychological skills and resources typically required to cope with stressors and outcomes of this magnitude (e.g., Zunin and Myers, 2000). Athletes who had good prior mental health were perceived to be better equipped to deal with pandemic-related consequences, while athletes with previous mental health challenges were reported as being more vulnerable and experiencing coping difficulties. This observation is consistent with Pensgaard et al.’s 2021 finding that athletes who were able to maintain their daily life routine and had higher perceived ability to cope with the crisis had fewer mental health symptoms. To date, studies conducted on the mental health of athletes during the pandemic were mostly quantitative, using transversal designs to assess the prevalence of mental health disorder symptoms at one time point during the pandemic (Jia et al., 2022). Only a few studies have compared mental health prior to and during the pandemic. Overall, these studies have shown a decline in athletes’ mental health during restrictive periods and lockdowns as well as an attenuating effect of being able to pursue training and have less restrictive socio-sanitary measures (Jia et al., 2022).

Other facilitative factors identified by the participants pertained to social support, mental performance skills, and communication. More specifically, it seemed that being able to preserve a sense of connectiveness with coaches and teammates through virtual communication was important for athletes’ social well-being, a result also observed by Elliott et al. (2021) who surveyed young athletes. Graupensperger et al. (2020) also found that university student-athletes who received more social support and experienced more connectedness with teammates during the pandemic reported better mental health. As depicted in the psychosocial phases of disaster model (Zunin and Myers, 2000), social support and communication are vital during any type of crisis.

What stands out in the current study is the role that mental performance skills played prior to and during the pandemic, influencing the well-being of athletes and helping them cope with the pandemic. While it is unclear which skills were prioritized by the athletes to cope with the pandemic, it appears that resilience, stress management, and emotional regulation were emphasized by MPCs and mental health practitioners who participated in this study. These skills were included in the GMP-SP designed to help steer mental performance programs in high-performance sport contexts and assist athletes in maintaining their mental health and responses to adversity (Durand-Bush et al., 2022). The findings of the current study lend support to the GMP-SP by demonstrating the intricate and vital interplay between mental performance competencies and mental health. Given that effective mental performance skills transfer emerged as an important finding in this study, more attention should be paid by practitioners when teaching mental performance skills to expand the reach so that athletes are equipped to apply them in a variety of sport and life situations.

With regard to debilitative factors impacting athletes’ mental health, participants discussed isolation, fear of the virus, uncertainty about the future, disruption of daily routines, and online communication fatigue. Some of these factors were highlighted in other studies conducted with elite athletes as well as research carried out with the general population. For instance, Simons et al. (2021) longitudinally examined elite Australian athletes and similarly found that uncertainty about the future and the unavailability of training facilities were among the most cited stressors. These findings are not dissimilar to those from the general population. For instance, Zheng et al. (2021) found in their study with a North American population that the perceived threat to participants’ own well-being or that of a family member was associated with elevated depressive symptoms. They also reported that social isolation was independently associated with elevated depressive symptoms. This is in line with the current study’s findings wherein isolation and fear of virus were among the most cited factors impeding athletes’ mental health.

### Consequences of COVID-19 for athletes

In terms of consequences of the pandemic for athlete mental health, both positive and negative outcomes were discussed by participants. It is noteworthy that the pandemic generated mental health challenges and mental illness symptoms for some athletes. This is not surprising given the prevalence of mental health challenges and the risk for mental illness in high-performance sport (e.g., Schinke et al., 2017; Reardon et al., 2019), and the fact that the general population reported poorer mental health during the pandemic (see Abdalla et al., 2021). While MPCs play an important role in promoting and fostering mental health, most MPCs in Canada are not dually trained and as such, athletes must be referred to mental health practitioners if they are struggling. A strong network of practitioners that included both MPCs and mental health practitioners was created and made available to most Canadian national team athletes throughout the pandemic, which is in line with recommendations provided in the Strategy for High Performance Sport in Canada (Durand-Bush and Van Slingerland, 2021). This may help explain why some athletes appeared to have effectively coped with the pandemic, despite the difficult periods and factors they encountered.

While it is important to acknowledge the negative consequences of the COVID-19 pandemic on athletes’ mental health, our results support the idea that some athletes experienced the crisis as a welcoming break and opportunity for self-care. This finding emphasizes the importance of recovery and rest on performance and well-being (Kellmann et al., 2018). It also sheds light on the pressure and training overload experienced by elite athletes. Much like the general population, athletes need opportunities to take a step back from their work and career. We also found that having time for life outside of sport was a facilitative consequence of the pandemic. Similarly, in a study with Olympic and Paralympic Norwegian athletes, Pensgaard et al. (2021) found that some athletes perceived positive consequences related to the COVID-19 pandemic and had fewer mental health symptoms and a greater level of satisfaction with life, which is an important dimension of emotional well-being (Westerhof and Keyes, 2010). Furthermore, in a qualitative study examining the perceptions of Austrian Olympic athletes and coaches on the impacts of the Tokyo 2020 Olympic Games postponement, some participants perceived the postponement as an opportunity for extra time to train and recover from injuries (Oblinger-Peters and Krenn, 2020). Moreover, many reported benefitting from time to pursue other interests beyond sport and enjoy different aspects of life. Equally, Karipidis and Steinfeldt (2020) argued that social distancing can be used as an opportunity to explore new interests and enhance interpersonal relationships. Altogether, these results reinforce the importance to consider athletes as holistic individuals with their own personal needs beyond sport, who seek self-actualization in the multiple aspects of their lives.

### Impact of the pandemic on practitioners

MPCs and mental health practitioners described an increase in workload during the pandemic. They played a vital role in providing support to athletes, coaches, and support staff. Indeed, some athletes used the pandemic as an opportunity to work on their mental skills, both because of the time available and the necessity to better respond to situations. However, the role of MPCs shifted from mental performance to prepare for Olympic/Paralympic Games qualifications and competitions, to mental health (i.e., psychological, emotional, social well-being) to help athletes cope with the pandemic and emerging stressors. This finding is innovative as it has been unclear in the literature to what extent MPCs support the mental health of athletes. It corroborates recent evidence and calls for increased collaborative work between MPCs and mental health practitioners to ensure that athletes can sustain their well-being while striving to achieve their performance goals (Van Slingerland et al., 2019; Henriksen et al., 2020; Durand-Bush and Van Slingerland, 2021). It also demonstrates the leadership of MPCs and mental health practitioners in supporting national sport organizations and the expertise they bring to the overall athlete support team. Even though this study was conducted in the context of the COVID-19 pandemic, it is plausible to hypothesize that the work of MPCs and mental health practitioners may serve as a protective factor for other challenges faced by national team athletes such as injuries, deselection, maltreatment, and retirement to give a few examples. However, regular access to MPCs is often only available when athletes reach the elite or national level due to a lack of awareness of services offered by MPCs at lower levels in the sport system, a lack of availability of MPCs working at these lower levels, and/or a lack of personal or organizational financial support to cover the costs of these services. Consequently, this type of training should be integrated as early as possible in competitive sport settings. Athletes face many sporting and life challenges that they could more successfully overcome if they received proper mental health support throughout their sport career.

An area that warrants further investigation is the unique and respective contribution of MPCs and mental health practitioners and, most importantly, how these two types of practitioners can work collaboratively to optimize holistic care for athletes. The focus brought on the mental health of athletes during the pandemic seems to have raised awareness of the value of the work of MPCs and mental health practitioners within national sport organizations and integrated support teams. For many organizations, the unique area of competencies and scope of practice of MPCs and mental health practitioners is still misunderstood (McHenry et al., 2021). Notwithstanding this observation, collaborative care is a unique and proven feature of the mental healthcare model implemented within the Canadian Centre for Mental Health and Sport (CCMHS)—a national hub offering mental healthcare and resources to high-performance athletes, coaches, and support staff (Van Slingerland and Durand-Bush, 2021). Preliminary evidence supports the integration of MPCs and mental health practitioners (i.e., psychologists, counselors, psychotherapists, and social workers) when working with athletes (Van Slingerland and Durand-Bush, 2021); however, more research is warranted. With accruing evidence demonstrating the interplay between mental health and mental performance in both the current study and the literature (e.g., Durand-Bush and Van Slingerland, 2021; Durand-Bush et al., 2022), it is important to continue investigating and promoting the collaboration between experts in these domains.

### Strengths, limitations, and future recommendations

The current study has unique strengths and addresses gaps in the current literature. The primary strength of the current study is the breadth of the research questions. Questions addressed both the mental health and mental performance of Canadian high-performance athletes and the interplay between these variables. The current study also examined factors impacting mental health during the pandemic as well as consequences, while most studies have focused on outcomes of the pandemic. Further, these factors and consequences were not limited to debilitative or negative aspects of the pandemic, but also facilitative and positive ones. Finally, the current study included MPCs and mental health practitioners, which provided a different voice and further insight into athletes’ experiences. It allowed for the exploration of the role these individuals played during the initial months of the pandemic and additional resources required to better address major disruptions in the future.

Limitations of the current study include the timing of the focus group interviews and varying levels of COVID 19-related restrictions across Canada. Due to these constraints, some participants were not able to participate in the focus groups and participated in an individual interview instead, which may have influenced the information they provided (e.g., they did not benefit from a collective discussion with peers). During the second wave of the pandemic when data collection occurred, some provinces were under much stricter mandates due to greater cases and hospitalizations, while other provinces were not as affected and had returned to close-to-normal sport training and participation. Our sample included participants from multiple provinces, and as such, their experiences and perceptions were likely affected by these different restrictions. Future pandemic-related research should examine fluctuations in mental performance and mental health based on different restrictions and sport environments that may be affected by varying constraints. Finally, while the sample included participants who worked with over 30 Canadian sports/teams, the size was still relatively small and more women than men participated. Future studies should aim to include a larger sample comprised of both practitioners and athletes from different genders to triangulate perceptions and experiences.

## Conclusion

This study was one of the first to examine the mental health and mental performance of athletes from the perspective of MPCs and mental health practitioners. Both positive (i.e., social support, communication, good prior mental health, and mental performance) and negative (e.g., fear, isolation, and stress) factors impacted athletes’ mental health during the COVID-19 pandemic. More specifically, athletes with well-developed mental performance skills prior to the pandemic were perceived to have better mental health during the pandemic and to be more equipped to cope with debilitative consequences of the pandemic than those who had poor mental performance skills and poor mental health at the onset of the global crisis. MPCs and mental health practitioners played a key leadership role in supporting national/Olympic/Paralympic athletes and national sport organization during the pandemic. The mental performance skills developed by the athletes during the pandemic with the support of MPCs and mental health practitioners facilitated their psychological, emotional, and social well-being. The pandemic had both debilitative and facilitative consequences on athletes’ mental health. For some athletes, it generated unpleasant emotions (e.g., low mood and anxiety), while for others, it exacerbated existing symptoms of mental illness. However, the pandemic provided many athletes with a welcomed break from training and competition and allowed them to rest and enjoy their life outside of sport.

From an organizational standpoint, there is an overall need for more mental health resources and better access to MPCs and mental health practitioners in the Canadian sport system. By examining the interplay between mental performance and mental health in high-performance sport and demonstrating that greater mental skills may help athletes better cope with both sport and non-sport-related stressors (e.g., global pandemic), it seems that sport organizations should prioritize mental skills training as early as possible in athletes’ career and allocate resources for these types of services. Furthermore, given the benefits resulting from engaging in additional rest and recovery during the pandemic, sport leaders should carefully examine common norms in high-performance sport that “doing more” results in better results. Taking time away from sport and exploring other areas of their life may be tremendously valuable to elite athletes in the long-term.

## Data availability statement

The raw data supporting the conclusions of this article will be made available by the authors, without undue reservation.

## Ethics statement

The studies involving human participants were reviewed and approved by Dalhousie University Research Ethics Board. The patients/participants provided their written informed consent to participate in this study.

## Author contributions

LD was responsible for the conceptualization of the research, contributing to the research design, participant recruitment, and data analysis, and was a primary contributor to the writing of the manuscript. VB and ND-B contributed to the conceptualization of the research and its design, the data analysis, and also primary contributors to the writing of the manuscript. LM was primarily responsible for participant recruitment, data collection and data analysis, and contributed to the writing of the manuscript. VG was responsible for the data collection that occurred in French and for the data analysis and contributed to the writing of the manuscript. All authors contributed to the article and approved the submitted version.

## Funding

Funding for this study was provided by the Social Sciences and Research Council of Canada’s (SSHRC) Partnership Engage Grant, in partnership with Own The Podium (grant #1108-2020-1024).

## Conflict of interest

The authors declare that the research was conducted in the absence of any commercial or financial relationships that could be construed as a potential conflict of interest.

## Publisher’s note

All claims expressed in this article are solely those of the authors and do not necessarily represent those of their affiliated organizations, or those of the publisher, the editors and the reviewers. Any product that may be evaluated in this article, or claim that may be made by its manufacturer, is not guaranteed or endorsed by the publisher.
